# Influence of pleated geometry on the pressure drop of filters during dust loading process: experimental and modelling study

**DOI:** 10.1038/s41598-022-24838-7

**Published:** 2022-11-25

**Authors:** Guangping Teng, Guoqing Shi, Jintuo Zhu

**Affiliations:** 1 School of Safety Engineering, China University of Mining and Technology, Xuzhou, 221116 China; 2 School of Safety and Management Engineering, Hunan Institute of Technology, Hengyang, 421002 China; 3 State Key Laboratory of Coal Resources and Safe Mining, China University of Mining and Technology, Xuzhou, 221116 China

**Keywords:** Occupational health, Fluid dynamics

## Abstract

In this study, a pressure drop model was developed through numerical simulation and experimentation for optimizing the design of the pleated structure of the filter media to extend the service life of the filter and reduce the ventilation energy consumption. The effect of the Stokes number on the dust deposition on the pleated filter media was revealed through numerical simulation. On this basis, a pressure drop model during dust loading was developed. The model suggests that under the same mass of dust deposition per unit area (*W*), the greater the pleat ratio (α), the greater the dust cake thickness and the effective filtration velocity in the effective filtration area. In addition, for V-shaped and U-shaped filters, the relative mean deviations between experimental and modeling total pressure drops are 3.68% and 4.82%, respectively. In other words, the proposed model accurately predicts pressure drop during dust loading. Furthermore, under the same α and $W$, the total pressure drop of the U-shaped filter is lower than that of the V-shaped filter, demonstrating the superior filtration performance of the U-shaped filter.

## Introduction

Pleated filters are commonly used in the fields of HVAC systems and air purifiers. Through pleated filter media, A larger filtration area and lower filtration velocity can be achieved in limited space, extending the filter replacement period^1–7^. Filtration efficiency and pressure drop are two important indicators of filter performance. Filtration efficiency is heavily influenced by filter media and filtration velocity. The pleat geometry has a limited effect on the filtration efficiency^8–10^. The pleat-induced bending deformation of the filter media, on the other hand, will result in changes in its permeability and porosity, increasing in the pressure drop of the filter^3,11–13^. Meanwhile, the pleated geometry will cause variation in the airflow field and non-uniform dust deposition, and thus the pressure drop during dust loading is greater than that of flat filtration^12,14–18^. The pleated geometry has a significant impact on the filter performance. However, current researches on pressure drop prediction during dust loading are insufficient, and the majority of them are experimental studies^19–22^.

At present, studies are being conducted to predict the pressure drop of filters with various pleated geometries^1,11,14–16,23–26^. Caesar et al.^15^ developed a pressure drop prediction model for clean V-shaped and U-shaped filters, dividing the total pressure drop into three components: pressure difference inside the pleat due to friction losses and dynamic pressure gain; pressure drop due to contraction and expansion when air enters and exits the pleat system; and pressure drop when air flows through the filter media. The filtration velocity in their study ranges from 1 to 10 m/s, and the ratio of the pressure drop caused by the pleated geometry to the total pressure drop grows as filtration velocity increases. Del Fabbro et al.^26^ established a semi-empirical dimensionless model that predicted the pressure drop of clean filters based on filter media type, pleated geometry, and flow parameter. However, the preceding studies were limited to clean filters and did not include the prediction of pressure drop during dust loading.

The prediction of pressure drop of filters with different pleated geometries during dust loading has rarely been reported so far. Fotovati et al.^18^ investigated non-uniform dust deposition on V-shaped and U-shaped filters and calculated pressure drop variation with dust deposition. Nonetheless, the dust particles in their study were 3 μm and 10 μm in size, whereas in reality the dust particles are mostly polydisperse. Using the analytical expressions for the x and y components of the velocity field inside V-shaped and U-shaped channels, Saleh et al.^27^ deduced a simple semi-numerical model that can be applied to predict the pressure drop of pleated filters during dust loading. However, the model does not consider the uneven dust deposition on the pleated area of the filters.

Through investigating the airflow fields and PM_10_ deposition of V-shaped and U-shaped filters, a pressure drop model during dust loading was established in this study, which can be used to optimize the design of the pleated structure of the filter so as to reduce the resistance, thus extending the service life of the filters and reducing the ventilation energy consumption.

## Research method

### Pleated filters

**Figure 1:**
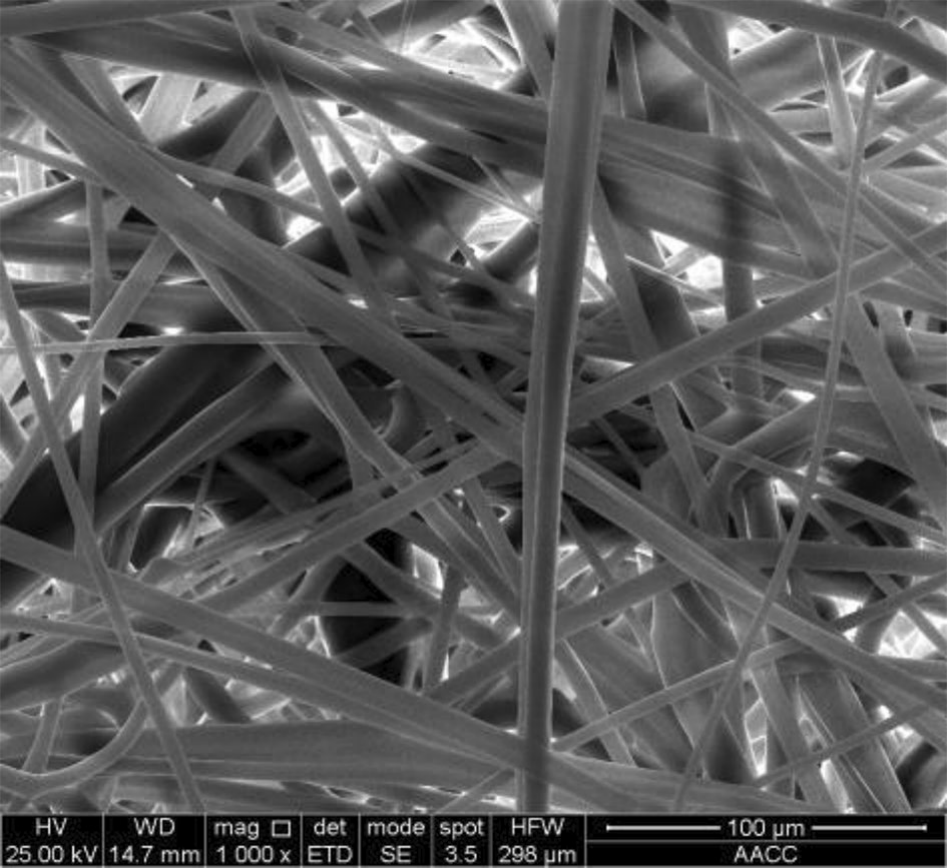
SEM image of the filter media under the magnification of 1000.

The polypropylene microfiber (Henan Aklly Filter Engineering Co., Ltd., Xinxiang, China) was selected as the filter media, which has a filter grade of E10 (classified according to EN 1822-1-2019^28^), a thickness of 0.5 mm, and a grammage of 110 g/m^2^. The SEM (VEGA, TESCAN, Czech) image of the filter media is presented in Fig. 1, and the fiber structure shows a three-dimensional disordered spatial network. According to statistical calculation of the fiber diameter in the image, the average diameter of the filter fiber was around 4.6 ± 0.3 μm.

**Figure 2:**
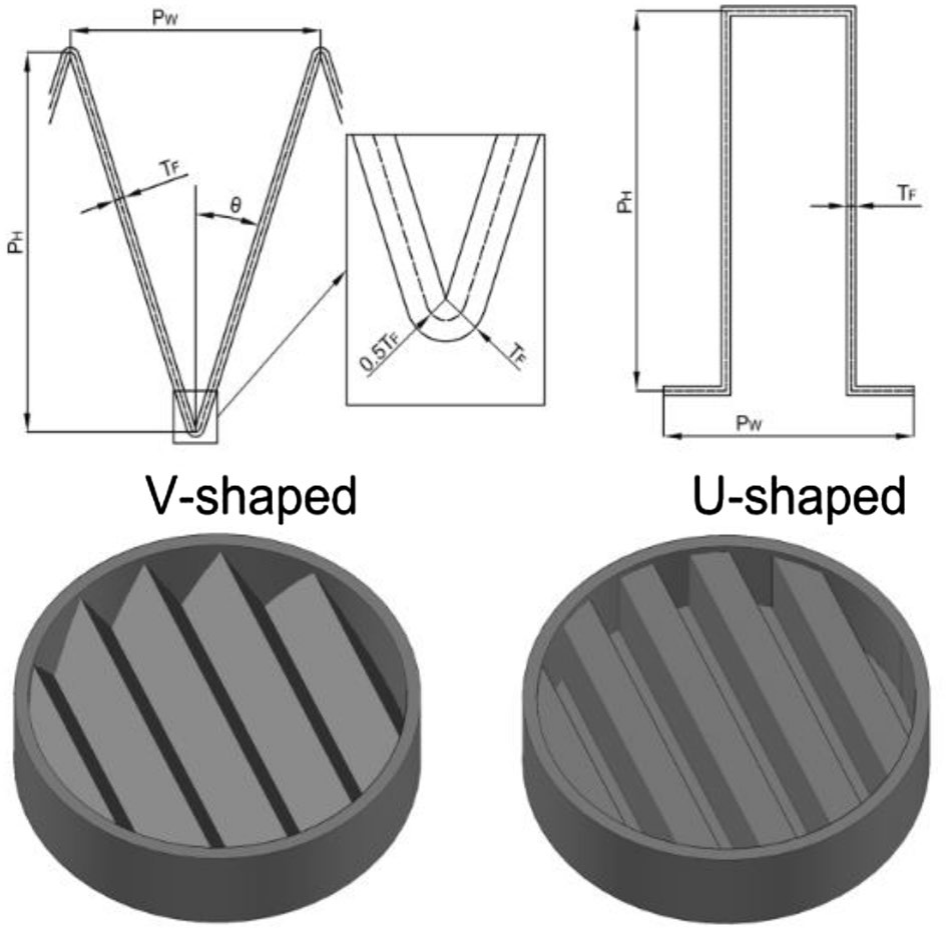
Filters with different geometries.

The pleated filters used in experiment were handcrafted. A transparent polymethyl methacrylate ring with an outer diameter of 150 mm, an inner diameter of 140 mm, and a height of 25 mm served as their outer frame. The geometries of the V-shaped and U-shaped filters are depicted in Fig. 2. The number of pleats of the handcrafted filter was 5, 10, 15, 20, and 25, respectively, and the pleat height was 20 mm. A hot-melt adhesive was used to secure the filter media to the outer frame. The pleated areas needed to be simplified with a certain thickness of filter media. The pleated area in the V-shaped filter was an arc with a radius equal to the thickness of the filter media ($T_{F}$) in the outer part of the pleat corner. The pleated area in the U-shaped filter was regarded as a right angle. The central location of filter media was used to calculate the parameters of filters with different geometries (the dotted line in Fig. 2).

The research for the V-shaped filter was conducted on an individual pleat.

1 $$\tan \theta = \frac{{0.5P_{W} - T_{F} \cos \theta }}{{P_{H} - T_{F} + T_{F} \sin \theta }}$$

where θ is the pleat angle (°), $P_{W}$ is the pleat pitch (m), $T_{F}$ is the thickness of filter media (m), and $P_{H}$ is the pleat height (m). As a result, the values of θ under various pleat ratios (α) can be calculated. The theoretical filtration area per unit length of an individual pleat can be expressed as:

2 $$\begin{gathered} s_{Vt} = 2\left( {\frac{90 - \theta }{{180}}\pi T_{F} + \frac{{P_{H} - T_{F} + T_{F} \sin \theta }}{\cos \theta }} \right) = 2(s_{Vt1} + s_{Vt2} ) \hfill \\ s_{Vt1} = \frac{90 - \theta }{{180}}\pi T_{F} \hfill \\ s_{Vt2} = \frac{{P_{H} - T_{F} + T_{F} \sin \theta }}{\cos \theta } \hfill \\ \end{gathered}$$

where $s_{Vt}$ is the theoretical filtration area per unit length of an individual V-shaped pleat (m^2^); $s_{Vt1}$ is the arc area per unit length of pleat corner (m^2^); and $s_{Vt2}$ is the inclined plane area per unit length of the pleat (m^2^). When the filter is circular with a diameter (*D*) of 140 mm and N pleats, the theoretical filtration area ($S_{V}$) is given as:

3 $$S_{V} = \frac{{\pi Ns_{Vt} D}}{4}$$

In the case of an individual pleat, the theoretical filtration area per unit length for the U-shaped filter is

4 $$s_{Ut} = 2P_{H} + P_{W}$$

The theoretical filtration area of a circular filter with a diameter (D) of 140 mm is expressed as:

5 $$S_{U} = \frac{{\pi Ns_{Ut} D}}{4}$$

The pleat angle θ in the V-shaped filter is obtained by Eq. (1), and the theoretical filtration area of filters with different geometries can be obtained by Eqs. (3) and (5). The specific parameters are listed in Table 1, where the pleat ratio of the flat filter is 0.

**Table 1:** Parameters of the self-made pleated filters.

Pleat ratio (α)	0	0.71	1.43	2.14	2.86	3.57
Number of pleats (N)	–	5	10	15	20	25
Pleat height P_H_ (mm)	–	20	20	20	20	20
Pleat width P_W_ (mm)	–	28	14	9.3	7	5.6
Pleat ratio α (P_H_/P_W_)	0	0.71	1.43	2.14	2.86	3.57
Pleat angle of the V-shaped filter θ (°)	–	34.48	18.36	12.03	8.73	6.72
Filtration area of the V-shaped filter S_V_ (cm^2^)	153.9	261.7	461.9	676.6	895.5	1116.2
Filtration area of the U-shaped filter S_U_ (cm^2^)	153.9	351.7	571.5	791.3	1011.1	1230.9

### Particulate matters

**Figure 3:**
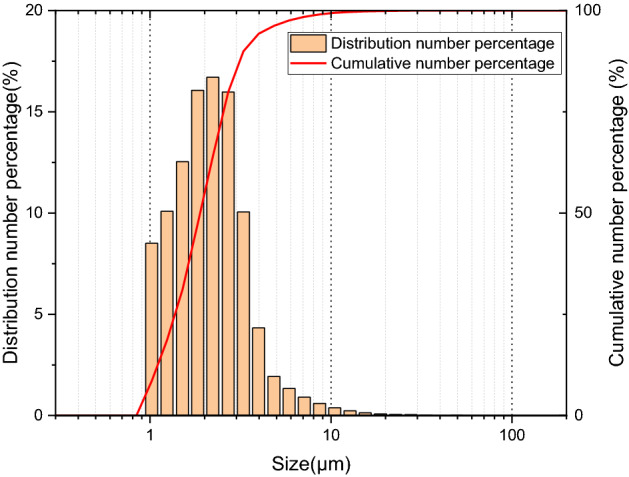
Particle size distribution of dust.

This experiment used 400-mesh fly ash with a density of 620 kg/m^3^ that was dried 100 °C for 5 h. A laser particle size analyzer (Winner2000, Jinan Winner Particle Instrument Stock Co., Ltd., Jinan, China) was used to measure the particle size distribution, as presented in Fig. 3. The count average particle size and count median particle size of the dust particles are 2.39 μm and 1.98 μm, respectively.

### Simulation

**Figure 4:**
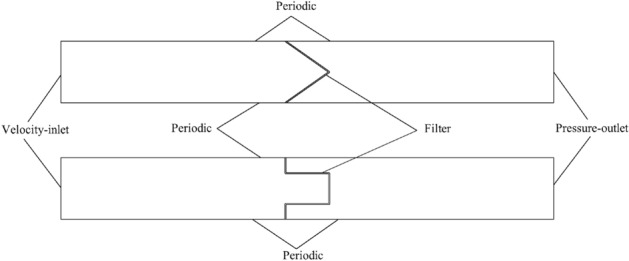
Numerical simulation computational region.

Two-dimensional numerical simulation was adopted in this study to explore the effect of pleated geometry on the airflow field and dust particle motion trajectory, and the computational region and boundary conditions are depicted in Fig. 4. The computational region is divided into three zones: the pleated filter media zone, the upstream velocity field zone of the filter, and the downstream velocity field zone of the filter (Fig. 4). Among these three zones, the pleated filter media zone is an isotropic porous media area where the performance of the filter media bending zone is ignored. The upstream and downstream velocity field zones of the filter are both 100 mm long. The velocity inlet and the pressure outlet are on the left and right sides, respectively, and the upper and lower sides are periodic boundaries that serve to reduce the influence of the boundary layer on the velocity field. The computational region is subject to unstructured meshing, with mesh sizes of 0.05 mm and 0.2 mm for the porous media zone and other zones, respectively. Based on this, the computational domains of the V-shaped and U-shaped filters under different α values are meshed. The pressure drops and velocity distribution in finer meshes are essentially the same as the values obtained here, indicating that the meshing in this study is reasonable.

In this study, ANSYS Fluent 19.0 software was used for the numerical simulation of the V-shaped and U-shaped filters with varying α values. The inlet air velocities of V-shaped and U-shaped filters under different α values when the filtration velocity is 4 cm/s are depicted (Table 2). Because the Reynolds number is small, laminar simulation is applied. The values of the parameters in the numerical simulation are as follows: the air density is 1.2 kg/m^3^, the dynamic viscosity is 1.8156 × 10^−5^ Pa·s, the permeability is 9.5810 × 10^−12^ m^2^, and the porosity is 0.84. The gauge pressure is set to 0 Pa at the outlet, and pressure–velocity coupling is performed using the SIMPLEC algorithm.

**Table 2:** Inlet air velocities when the filtration velocity is 4 cm/s.

Filter	α = 0.71	α = 1.43	α = 2.14	α = 2.86	α = 3.57
Inlet airflow velocity for the V-shaped filter v_i_ (cm/s)	6.80	12.00	17.57	23.26	28.99
Inlet airflow velocity for the U-shaped filter v_i_ (cm/s)	9.14	14.86	20.57	26.29	32.00

### Experimental system and procedure

**Figure 5:**
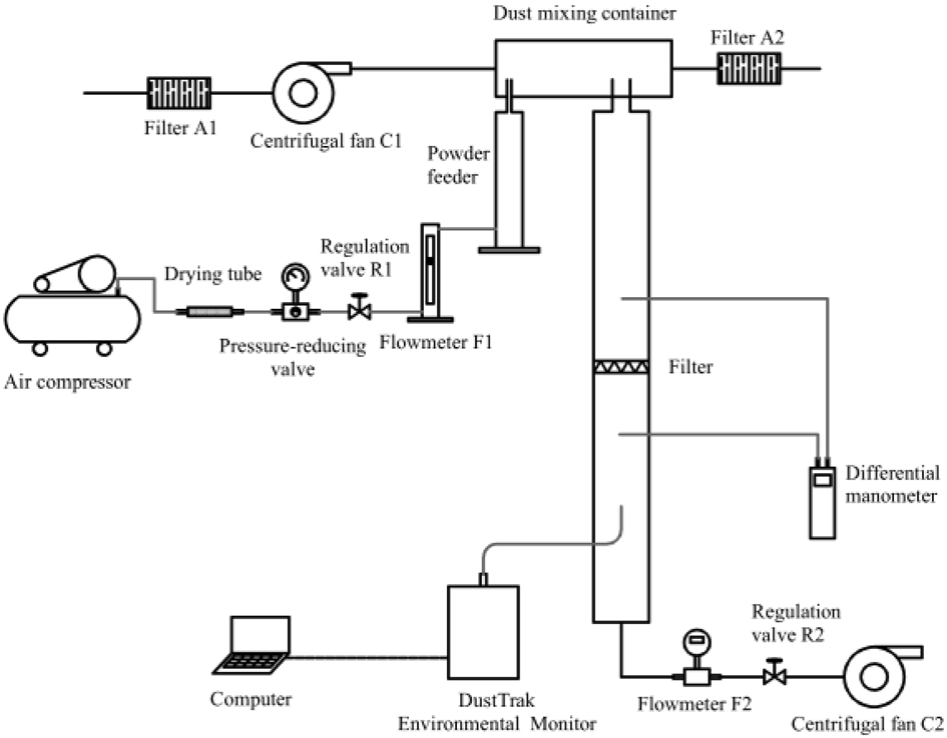
Schematic diagram of the experimental system.

The dust filtration experimental system, as illustrated in Fig. 5, is made up of three parts: the dust generation unit, the filtration unit, and the monitoring unit. An air compressor, a drying tube, a pressure-reducing valve, a flow regulation valve R1, a flowmeter F1, a powder feeder (Solid particle generator 9309, TSI, the USA), a dust mixing container, and a centrifugal fan C1 constitute the dust generation unit. The procedure of dust generation is described as follows: first, the high-pressure air from the air compressor was adjusted to a low and constant pressure airflow via the drying tube and the pressure-reducing valve; the airflow was then adjusted via the flow regulation valve R1 to control the supply of the powder feeder; the centrifugal fan C1 then provided constant airflow to dilute the dust in the dust mixing container and maintain its concentration constant. An air filter, a filter tube, a flow regulation valve R2, a centrifugal fan C2, and a flowmeter F2 were all part of the filtration system. The filter tube was composed of two sections of pipes with lengths of 400 mm, outer diameters of 150 mm, and inner diameters of 140 mm. The two sections of pipes clamped the filter through the connecting device. The monitoring unit included a differential manometer (AP800, TSI, the USA), and a DustTrak environmental monitor (8543, TSI, the USA) for measuring filter pressure drop and dust concentration. The maximum measurement of the manometer is 15 inH_2_O with the resolution of 0.001 inH_2_O and the error of less than 1%. The DustTrak environmental monitor 8543 can monitor PM_1.0_, PM_2.5_ and total dust concentrations.

The experiments were conducted in both a dust-free environment and a dust-containing environment. The filtration velocity in the clean airflow was set to 4 cm/s, and the flow was adjusted using the flow regulation valve R2. Specific flow data are listed in Table 3. The change in the pressure drop of the filter with flow under different α values was measured. The supply of dust in the dust-containing environment was adjusted by controlling the powder feeder, which kept the dust concentration in the dust-mixing container stable at 800 ± 50 mg/m^3^. Specific experimental procedure is as follows: the regulation valve R2 was first adjusted to change the filtration flow so that the average filtration rate stabilized at 4 cm/s; after that, the filtration duration was controlled until the mass of dust deposition per unit area reached 5 mg/cm^2^, 10 mg/cm^2^, 15 mg/cm^2^, 20 mg/cm^2^, 25 mg/cm^2^, and 30 mg/cm^2^, and the pressure drop was recorded; finally, the filter was weighed before and after each experiment, and the mass of dust deposition per unit area was calculated. The error had to be kept under 5% or the experiment had to be restarted. The above experiment was repeated three times to ensure its repeatability.

**Table 3:** Flow rates when the filtration velocity is 4 cm/s.

Filter	α = 0	α = 0.71	α = 1.43	α = 2.14	α = 2.86	α = 3.57
Flow rate of V-shaped filter (L/min)	37.0	62.8	110.9	162.4	214.9	267.9
Flow rate of U-shaped filter (L/min)	36.9	84.4	137.2	189.9	242.7	295.4

## Model

### Numerical simulation of different pleat geometries

**Figure 6:**
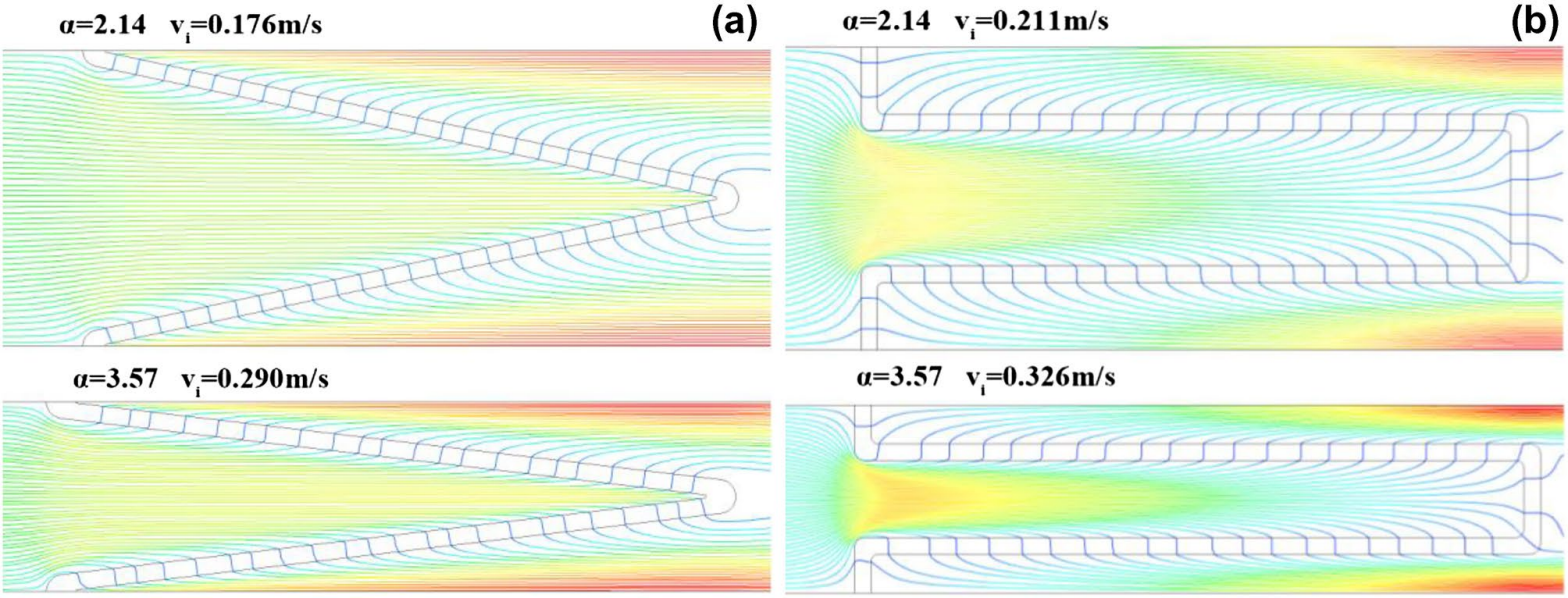
Airflow fields at the filtration velocity of 4 cm/s.

According to the method described in Section “Simulation”, the airflow fields of V-shaped and U-shaped filters under different α at the filtration velocity of 4 cm/s was obtained using numerical simulation. It was found that velocity of air flowing through filter media exhibits the same variation trend, that is, the airflow velocity of filter media in the pleated area is low, while those in other areas are essentially the same. The airflow fields under α values of 2.14 and 3.57 are illustrated in Fig. 6. The permeability of the filter media used in the numerical simulation is assumed to be constant. Because the bending deformation of the filter media may limit the permeability, the real airflow velocity of the filter media in the pleated area is lower than that in the numerical simulation. Furthermore, some scholars^14,16,17^ reported that the airflow velocity of filter media in the pleated area is incredibly low, and the simulated pressure drop obtained by non-permeability treatment is more accurate than that acquired by experiments. Therefore, the pleated area is regarded as a non-permeable, ineffective filtration area in this study.

According to current research findings, dust deposition on the filter media is closely correlated with Stokes number ($S_{tk}$)^27,29,30^. The Stokes number can be expressed as

6 $$S_{tk} = \frac{{\tau u_{0} }}{{L_{0} }} = \frac{{\rho_{p} d_{p}^{2} u_{0} }}{{18\mu L_{0} }}$$

where $\tau$ is the relaxation time of particle (s), $u_{0}$ is the velocity of particles flowing through obstacles (m/s), $L_{0}$ is the characteristic quantity of obstacles (m), $\rho_{p}$ is the particle density (kg/m^3^), $d_{p}$ is the particle diameter (m), and $\mu$ is the dynamic viscosity of air (Pa·s). Equation (6) exhibits that the higher the velocity and larger the dust particle size, the greater the $S_{tk}$. For the V-shaped and U-shaped filters, $L_{0}$ can be $P_{W}$ and $0.5\;P_{W}$, respectively. Under the same filtration velocity, $S_{tk}$ reaches its maximum value when α is 3.57.

**Figure 7:**
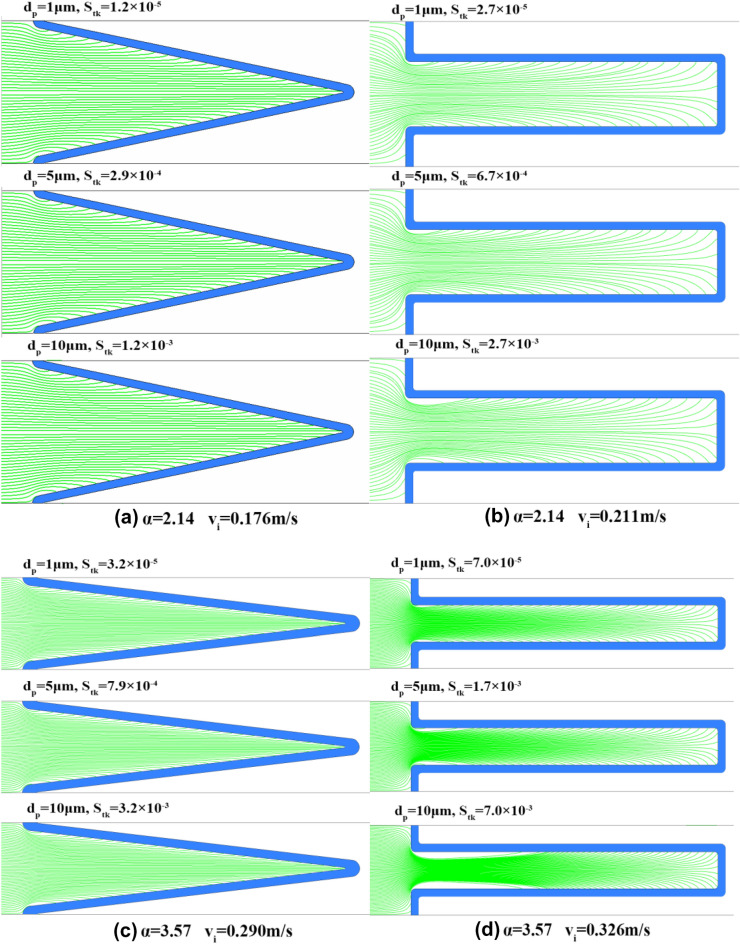
Motion trajectories of different-sized dust particles.

Using the discrete phase model, the motion trajectories of 1 μm, 5 μm and 10 μm particles were numerically simulated. It was found that dust deposition on the filter media changes in the same trend with the variation of the $S_{tk}$. The deposition of different-sized dust particles on the V-shaped and U-shaped filters under the α values of 2.14 and 3.57 are presented in Fig. 7. With the exception of the pleated area, dust deposition on the filter media for the V-shaped filter is essentially uniform and does not change with the $S_{tk}$ (see Fig. 7a,c). Few dust particles are deposited on the pleated area of the U-shaped filter. Additionally, outside of the pleated area, dust deposition on filter media varies with the $S_{tk}$, and the greater the $S_{tk}$, the more uneven the dust deposition (see Fig. 7b,d). This finding is consistent with the finding reported by Saleh et al. that for the U-shaped filter, dust deposition can be treated as uniform when the $S_{tk}$ is below 0.1, while when $S_{tk}$ is greater than 0.1, the dust cake thickness grows linearly with the pleat depth^27,29^. It is concluded that with the increase of $S_{tk}$, the area where dust particles cannot reach expands (see Fig. 7b,d). For example, when $d_{p}$ is 5 μm, the area on the filter media where dust particles cannot reach is tiny, but when $d_{p}$ is 10 μm, the area accounts for nearly one third of the filter area (see Fig. 7d). Since approximately 97% of dust particles are less than 5 μm and the percentage of those larger than 10 μm is less than 1% (see Fig. 3), it can be roughly inferred that dust particles are uniformly deposited on the effective filtering area of the U-shaped filter.

### Pressure drop model of V-shaped filter during dust loading

**Figure 8:**
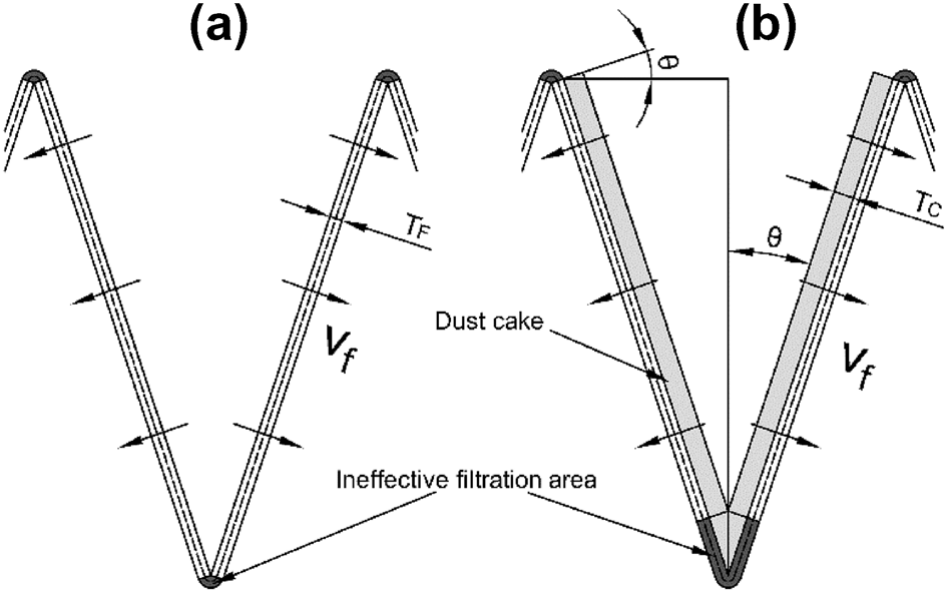
Dust deposition on the V-shaped filter.

According to the simulation results in Section “Numerical simulation of different pleat geometries”, the V-shaped pleat corner can be considered as an ineffective filtration area, as indicated in the black part of Fig. 8a. The remaining areas are effective filtration areas with the same effective filtration velocities, where receive an even deposition of dust particles. As the dust cake thickness increases caused by the dust deposition at the bottom pleated corner (Fig. 8b), the ineffective filtration area expands.

According to Eq. (2), $s_{Vt} = 2(s_{Vt1} + s_{Vt2} )$, the mass of dust deposition of per unit length of one individual V-shaped pleat can be stated as:

7 $$m_{V0} = 2(s_{Vt1} + s_{Vt2} )T_{C0} \rho = 2(s_{Vt1} + s_{Vt2} )W$$

where $m_{V0}$ is the mass of dust deposition (kg), $T_{C0}$ is the average dust cake thickness (m), $\rho$ is the bulk density of dust (kg/m^3^), and $W$ is the mass of dust deposition per unit area (kg/m^2^). When the pleat corner of the V-shaped filter is ineffective, the mass of dust deposition is:

8 $$m_{V} = 2(s_{Vt2} + \frac{{T_{C} }}{\tan \theta })T_{C} \rho$$

where $m_{V}$ is the mass of dust deposition (kg) and $T_{C}$ is the thickness of dust deposition on the effective filtration area (m). $m_{V}$ is equal to $m_{V0}$. According to Eqs. (7) and (8),

9 $$\frac{1}{2\tan \theta }T_{C}^{2} - s_{Vt2} T_{C} + (s_{Vt1} + s_{Vt2} )\frac{W}{\rho } = 0$$

The dust thickness of the V-shaped filter on the effective filtration area can be obtained:

10 $$T_{C} = \tan \theta \left[ {s_{t2} - \sqrt {s_{t2}^{2} - \frac{2W}{{\rho \tan \theta }}(s_{Vt1} + s_{Vt2} )} } \right]$$

With the increase in the mass of dust deposition, the effective filtration area decreases and the effective filtration velocity increases (Fig. 8). The effective filtration velocity ($\nu_{f}$) can be calculated using the invariant filter air volume.

11 $$\begin{gathered} \nu (s_{Vt1} + s_{Vt2} ) = \left( {s_{Vt2} - \frac{{T_{C} }}{\tan \theta }} \right)\nu_{f} \hfill \\ \nu_{f} = \frac{{\nu (s_{Vt1} + s_{Vt2} )}}{{\left( {s_{Vt2} - \frac{{T_{C} }}{\tan \theta }} \right)}} \hfill \\ \end{gathered}$$

where $\nu$ is the filtration velocity (4 cm/s). Some scholars divided the total pressure drop into two parts, i.e., the pressure drop caused by the pleated structure and the pressure drop caused by the filter media and dust cake^2,15,23^. Some studies ignored the pressure drop of pleated structures, primarily because the structural resistance is much smaller than the filtration resistance at low filtration velocity^27,31–33^. Because of the low filtration velocity, the structure resistance can be ignored in this study. Accordingly, the pressure drop caused by filter media and dust cake is considered as the total pressure drop and can be calculated using Darcy’s law^31–34^.

12 $$\Delta P_{T} = \Delta P_{F} + \Delta P_{C} = \left( {\frac{{\mu T_{F} }}{{K_{F} }} + \frac{{\mu T_{C} }}{{K_{C} }}} \right)\nu_{f}$$

where $\Delta P_{T}$ is the total pressure drop of the filter (Pa), $\Delta P_{F}$ is the pressure drop caused by the filter media (Pa), $\Delta P_{C}$ is the pressure drop caused by the dust cake (Pa), $K_{F}$ is the permeability coefficient of the filter media (m^2^), $K_{C}$ is the permeability coefficient of the dust cake (m^2^), and $\nu_{f}$ is the effective filtration velocity (m/s).

### Pressure drop model of U-shaped filter during dust loading

**Figure 9:**
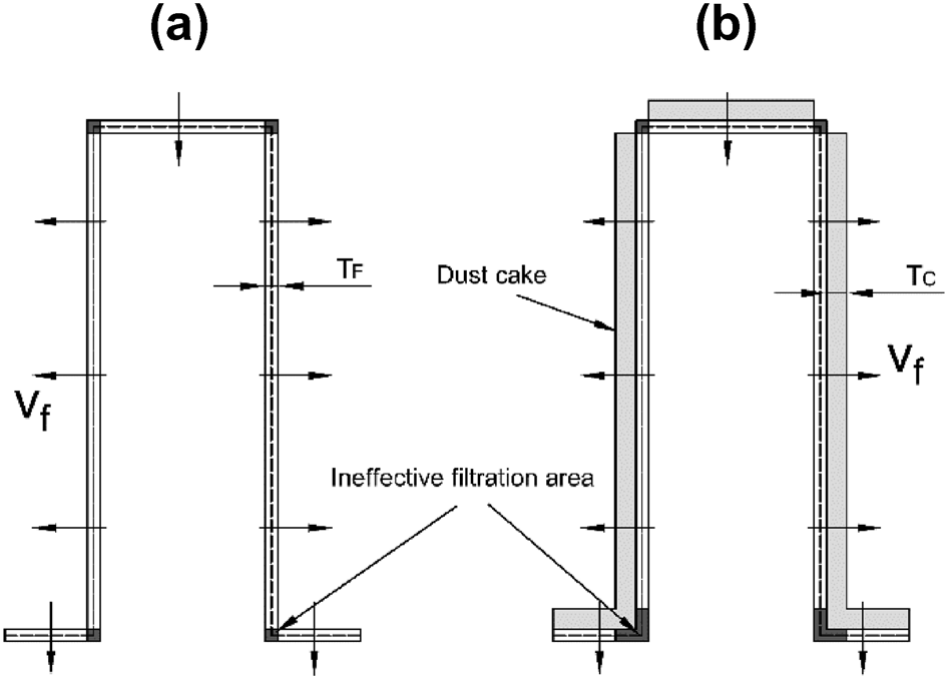
dust deposition on the U-shaped filter.

Consistent with the treatment method of V-shaped geometry described in Section “Pressure drop model of V shaped filter during dust loading”, the pleated area is considered as an ineffective filtration area, as illustrated in the black part in Fig. 9a. Except for the ineffective filtration area, all other areas are effective filtration areas with the same effective filtration velocities. Dust particles are uniformly deposited on the effective filtration area. Deposition at the lower pleated area causes the increase of the dust cake thickness, enlarging the ineffective filtration area (see Fig. 9b).

According to the Eq. (4), the theoretical filtration area per unit length of one individual U-shaped pleat can be expressed as $s_{Ut} = 2P_{H} + P_{W}$, thus the mass of dust deposition can be expressed as:

13 $$m_{U0} = (2P_{H} + P_{W} )T_{C0} \rho = s_{UT} W$$

When the pleated area of the U-shaped filter is ineffective, the mass of dust deposition is

14 $$m_{U} = (s_{Ut} - 4T_{F} - 4T_{C} )T_{C} \rho$$

$m_{V}$ equals $m_{V0}$. According to Eqs. (13) and (14),

15 $$4T_{C}^{2} - (s_{Ut} - 4T_{F} )T_{C} + s_{Ut} \frac{W}{\rho } = 0$$

The dust cake thickness of the U-shaped filter on the effective filtration area can be obtained:

16 $$T_{C} = \frac{1}{8}\left[ {s_{Ut} - 4T_{F} - \sqrt {(s_{Ut} - 4T_{F} )^{2} - 16s_{Ut} \frac{W}{\rho }} } \right]$$

With the increase in the mass of dust deposition, the effective filtration area declines, and the effective filtration velocity increases. According to the invariant filter air volume, the effective filtration velocity ($\nu_{f}$) can be obtained.

17 $$\begin{gathered} \nu s_{Ut} = (s_{Ut} - 4T_{F} - 4T_{C} )\nu_{f} \hfill \\ \nu_{f} = \frac{{\nu s_{Ut} }}{{(s_{Ut} - 4T_{F} - 4T_{C} )}} \hfill \\ \end{gathered}$$

The total pressure drop of the U-shaped filter can be calculated by Eq. (12).

## Results and discussion

### Changes in the permeability of dust cake during dust loading

**Figure 10:**
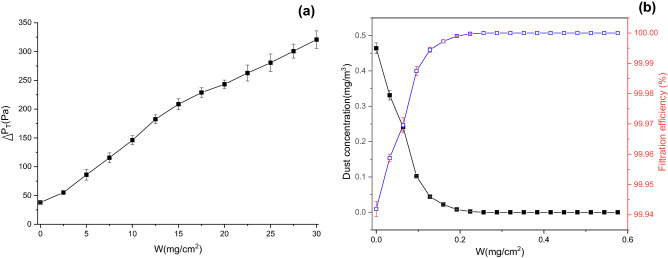
Changes in total pressure drop and dust removal efficiency with $W$ during plane filtration (**a**) change in total pressure drop with $W$, (**b**) changes in dust concentration and dust removal efficiency with $W$ after filtration.

The experimentally recorded change of total pressure drop and dust removal efficiency with the mass of dust deposition per unit area $W$ during plane filtration is depicted in Fig. 10. The dust concentration and the filtration velocity were set to 800 ± 50 mg/m^3^ and 4 cm/s, respectively. As presented in Fig. 10a, the total pressure drop increases as $W$ rises; besides, the increasing rate first climbs and then drops due to changes in the dust deposition characteristics. Song, Tanabe, and Li et al.^31,33,35^ found that the variation of pressure drop with dust deposition can be divided into three stages: depth filtration, transition, and surface filtration. Depth filtration occurs in the initial stage of filtration. At this stage, large particles deposit on the surface of the filter media, while fine particles enter into the filter media and get trapped, resulting in lower porosity of the filter media, and thus a slow but accelerated increase in pressure drop, as well as improved filtration efficiency. According to Fig. 10b, the dust removal efficiency rapidly rises from 99.9 to 100%. Then it enters the transition stage, during which a dust cake is formed on the surface of the filter media, indicating that depth filtration has been replaced by surface filtration, i.e., the third stage has begun. Surface filtration dominates the third stage, where the pressure drop increases linearly and the filtration efficiency reaches its peak. When *W* exceeds 15 mg/cm^2^, the total pressure drop rises approximately linearly, which is a mark of surface filtration; in contrast, when *W* is below 15 mg/cm^2^, it is in the depth filtration and transition stages (see Fig. 10a). In this study, dust particles are assumed to merely deposit on the surface of the filter media. Under such an assumption, the permeability coefficient of the dust cake can be calculated.

During flat filtration, the total pressure drop consists of two parts. i.e., the pressure drop caused by the filter media and the pressure drop caused by the dust cake. When $W$ is 0, the permeability coefficient of the filter media can be calculated by Eq. (12).

18 $$K_{F} = \frac{{\mu T_{F} \nu }}{{\Delta P_{F} }}$$

**Figure 11:**
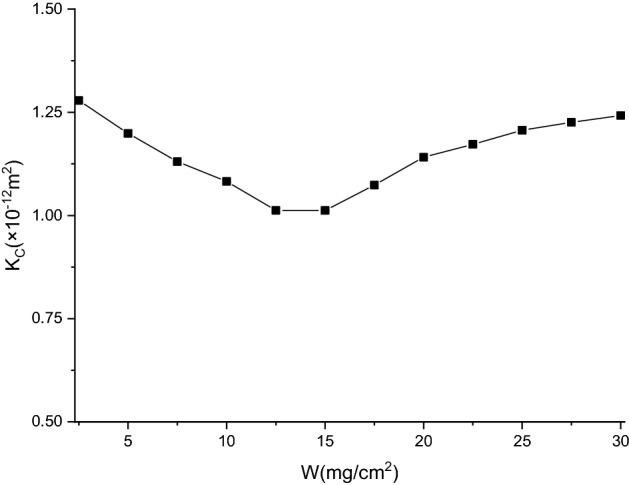
Change in permeability coefficient of dust cake with $W$ during flat filtration.

Based on the experimental conditions and results, $\Delta P_{F}$ is 37.9 ± 1.2 Pa, $\mu$ is 1.8156 × 10^−5^ Pa·s, $T_{F}$ is 0.0005 m, $\nu$ is 0.04 m/s, and $K_{F}$ is 9.5810 × 10^–12^ m^2^. When dust particles are deposited, $K_{C}$ can be expressed as

19 $$K_{C} { = }\frac{\mu W\nu }{{\rho (\Delta P_{T} - \Delta P_{F} )}}$$

where $\rho$ is the bulk density of dust (620 kg/m^3^). The change in the permeability coefficient of dust cake with $W$ is displayed in Fig. 11.

### Validation of pressure drop model

**Figure 12:**
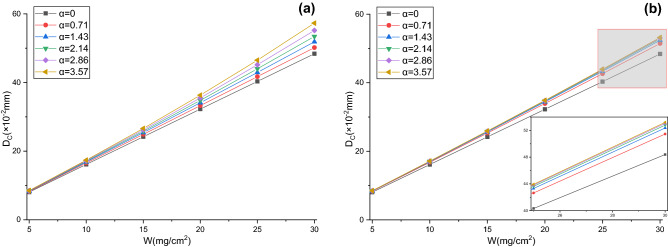
Dust cake thicknesses obtained by the model (**a**) V-shaped, (**b**) U-shaped.

**Figure 13:**
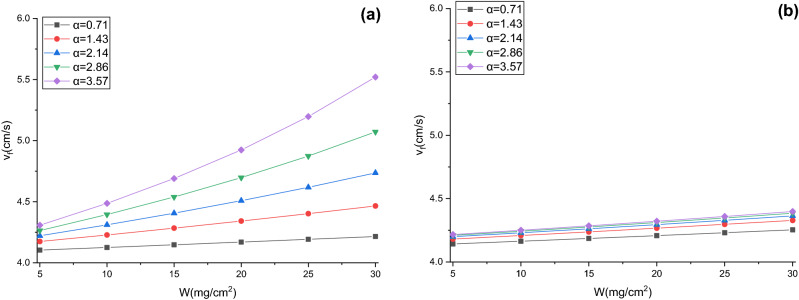
Effective filtration velocities obtained by the model (**a**) V-shaped, (**b**) U-shaped.

When $W$ are 5, 10, 15, 20, 25, and 30 mg/cm^2^, respectively, the thicknesses of dust cake deposited on the effective filtration area and the effective filtration velocities for V-shaped and U-shaped filters under different α can be calculated by Eqs. (10), (11), (16) and (17) (see Figs. 12 and 13). The thicknesses of dust cake for pleated filters obtained by the model are larger than those for flat filtration, and the thicknesses grow with the increases of α and $W$(see Fig. 12). The effective filtration velocities of pleated filters obtained by the model are higher than theoretical filtration velocities, and the velocities also increase with α and $W$ (see Fig. 13). This is due to the existence of ineffective filtration area in the pleated geometry, and the greater the α and $W$, the larger the ineffective filtration area, the greater the dust cake thickness and effective filtration velocity in the effective filtration area. The comparison between Fig. 12a,b indicates that under the same *W*, the dust cake thickness and effective filtration velocity in the effective filtration area of the V-shaped filter with different α differ significantly, whereas those in the U-shaped filter differ insignificantly. This is due to the rapid expansion of the ineffective filtration area caused by dust deposition at the pleat corner of the V-shaped filter.

**Figure 14:**
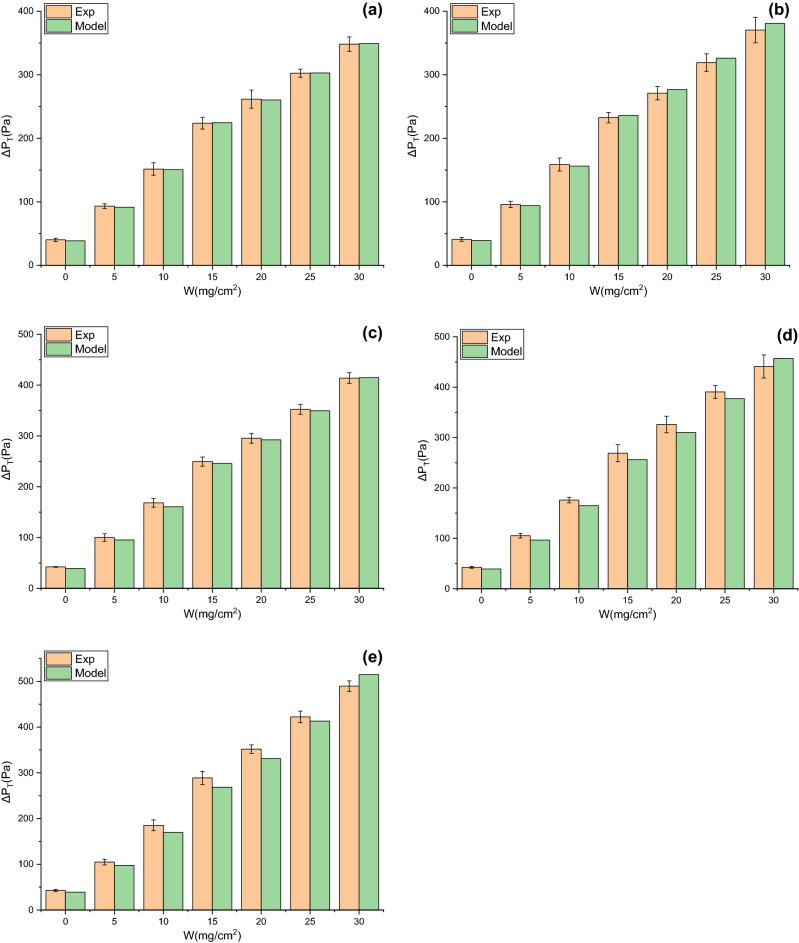
Changes in experimental and modeling total pressure drops with $W$ for V-shaped filter (**a**) α = 0.71, (**b**) α = 1.43, (**c**) α = 2.14, (**d**) α = 2.86, (e**)** α = 3.57.

**Figure 15:**
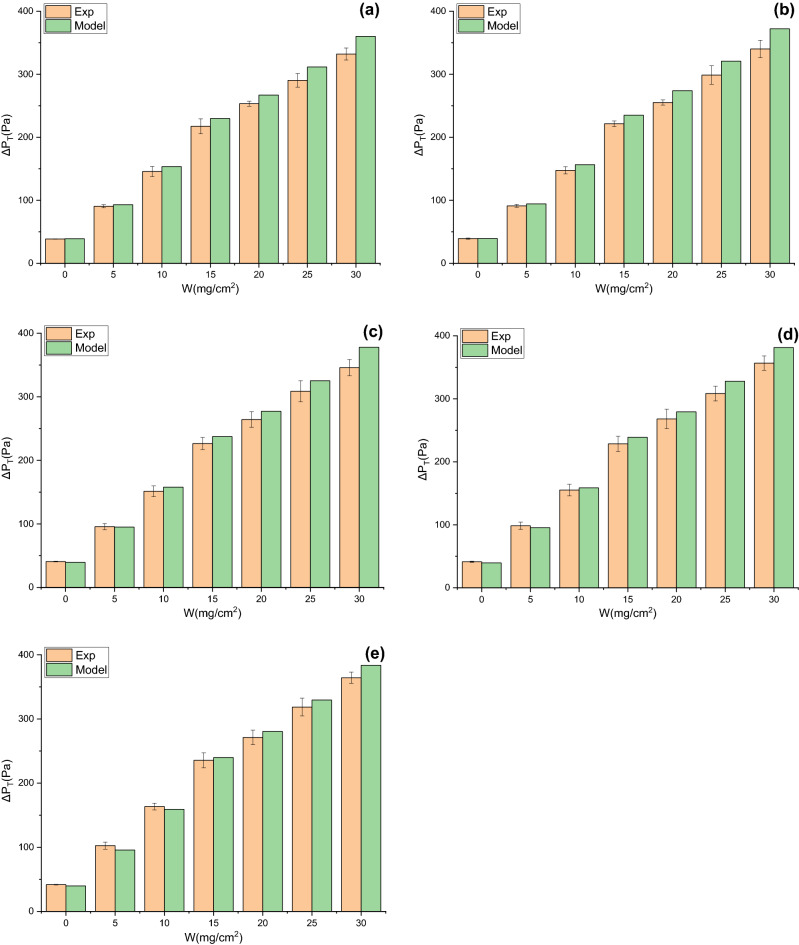
Changes in experimental and modeling total pressure drops with $W$ for U-shaped filter (**a**) α = 0.71, (**b**) α = 1.43, (**c**) α = 2.14, (**d**) α = 2.86, (**e**) α = 3.57.

Under the same $W$, compared with flat filtration, pleated filtration leads to the increases in the thickness of dust cake deposited on the effective filtration area and the effective filtration velocity, inevitably altering the permeability coefficient of the dust cake. However, it is difficult to measure the permeability coefficient of dust cake when the filter media are pleated. To calculate the total pressure drop by the model, it can be considered that under the same $W$, the permeability coefficient of dust cake during pleated filtration is the same as that during flat filtration. In this way, the total pressure drop of the model can be calculated through dust cake thickness and the effective filtration velocity obtained by Eq. (12). The changes in experimental and modeling total pressure drops with $W$ under different α for V-shaped and U-shaped filters are exhibited in Figs. 14 and 15, respectively. In the dust loading experiment, the dust concentration is 800 ± 50 mg/m^3^ and the theoretical filtration velocity is 4 cm/s. It can be seen that experimental and modeling total pressure drops for V-shaped and U-shaped filters present the same variation trend, both rising with the increases in α and $W$. Their relative errors range from − 8.17 to 5.15% and from − 6.72 to 9.42%, and their relative mean deviations are 3.68% and 4.82%, respectively. In conclusion, the proposed prediction model can accurately predict the total pressure drops of filters with different pleated geometries during dust loading. This also implies that it is reasonable to conduct non-permeability treatment for the pleated areas and to consume that dust particles are uniformly deposited on the effective filtration area. The data also reveals that under the same α and $W$, the total pressure drop of the U-shaped filter is lower than that of the V-shaped filter, demonstrating the superior filtration performance of the U-shaped filter.

**Figure 16:**
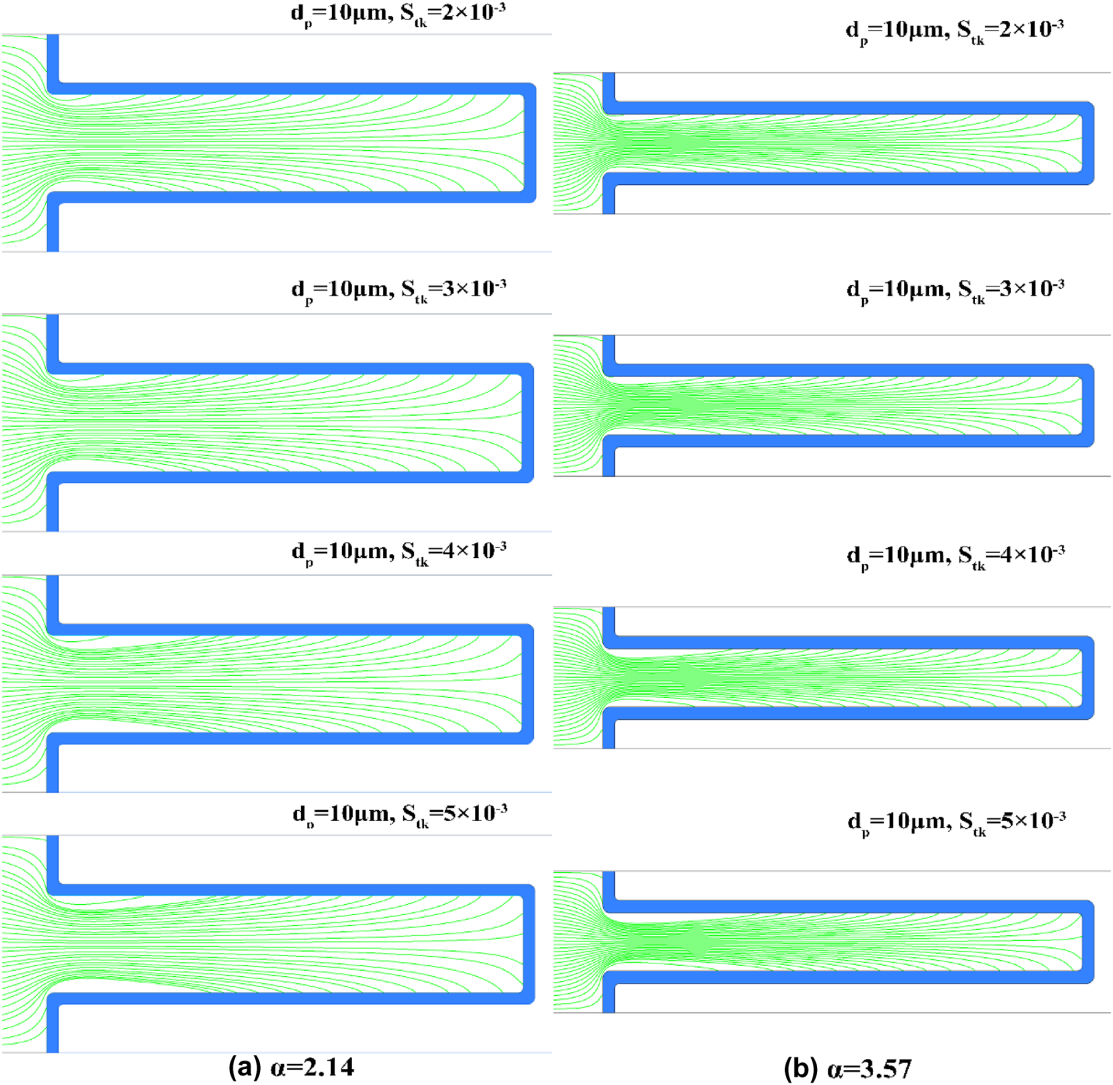
Motion trajectories of particles at different $S_{tk}$.

In Section “Numerical simulation of different pleat geometries”, the clean pleated filter media is divided into effective filtration area and ineffective filtration area according to the velocity of air flowing through filter media, which is the prerequisite for the model derivation. However, because dust loading alters the airflow field, the model is only applicable when the dust cake thickness is small. This study investigated particle size with diameters less than 10 μm. The experimental results show that the model can accurately predict the pressure drop when $W$ ≤ 30 mg/cm^2^, and the corresponding average dust cake thickness $T_{C0}$ is ≤ 0.48 mm. In practice, dust particles are unevenly deposited on the pleated filter media. In this study, there is no dust particles deposited on the ineffective filtration area, and dust particles are uniformly deposited on the effective filtration area, which is a simplified treatment when $S_{tk}$ is small. To determine the maximum $S_{tk}$ value applicable to the model, the motion trajectories of particles under different $S_{tk}$ were further explored through numerical simulation (see Fig. 16). It can be seen that the larger the $S_{tk}$, the more uneven the dust deposition on the effective filtration area. When $S_{tk}$ ≤ 3 × 10^−3^, dust particles can be considered to be uniformly deposited on the effective filtration area. Therefore, this model is applicable when the particle size is less than 10 μm, $S_{tk}$ ≤ 3 × 10^−3^ and $T_{C0}$ ≤ 0.48 mm.

## Conclusions

1. Through numerical simulation, the influence of Stokes number on the dust deposition on the pleated filter media was revealed. The deposition of dust particles on the effective filtration area of the V-shaped filter is essentially uniform. In contrast, for the U-shaped filter, the larger the $S_{tk}$, the more uneven the dust deposition on the effective filtration area.
2. A pressure drop model that can accurately predict the pressure drop of V-shaped and U-shaped filters during dust loading was put forward, which can be used to predict the service life of the filters. For V-shaped and U-shaped filters, the relative mean deviations between experimental and modeling total pressure drops are 3.68% and 4.82%, respectively.
3. Under the same α and $W$, the total pressure drop of the U-shaped filter is lower than that of the V-shaped filter, demonstrating the superior filtration performance of the U-shaped filter.

## Data Availability

The datasets used and/or analyzed during the current study are available upon reasonable request from the corresponding author.
